# Illness Anxiety Disorder: A Review of the Current Research and Future Directions

**DOI:** 10.1007/s11920-024-01507-2

**Published:** 2024-05-15

**Authors:** Katarina Kikas, Aliza Werner-Seidler, Emily Upton, Jill Newby

**Affiliations:** 1 Black Dog Institute, University of New South Wales, Hospital Road Randwick, Sydney, NSW 2031 Australia; 2 School of Psychology, University of New South Wales, Sydney, Australia

**Keywords:** Illness anxiety disorder, Hypochondriasis, Health anxiety, Anxiety disorders, Somatoform disorders, Diagnostic classification

## Abstract

**Purpose of Review:**

We review recent evidence on Illness Anxiety Disorder (IAD), including risk factors and precipitants, diagnostic classification, clinical characteristics of the disorder, and assessment and treatment in both children and adults.

**Recent Findings:**

IAD places a substantial burden on both individuals and society. Despite its impact, understanding of the disorder is lacking and debates remain about whether IAD should be classified as an anxiety disorder and whether it is distinct from Somatic Symptom Disorder. Cognitive behavioural therapy (CBT) is an effective treatment for IAD and there are multiple validated measures of health anxiety available. However, research on health anxiety in children and youth is limited.

**Summary:**

IAD is chronic, and debilitating, but when identified, it can be effectively treated with CBT. Research using DSM-5 IAD criteria is lacking, and more research is needed to better understand the disorder, particularly in children and youth.

## Introduction

While many individuals experience transient health concerns throughout their lives, a subset of individuals experience enduring and distressing anxiety about health and illness. Illness Anxiety Disorder (IAD) is a new disorder introduced in the latest version of the Diagnostic and Statistical Manual of Mental Disorders [DSM-5; 1], replacing the DSM-IV Hypochondriasis diagnosis. IAD is characterised by an intense fear of having or acquiring a serious medical disease or illness, such as cancer, heart disease or other significant conditions. Individuals with IAD experience illness-related cognitions, such as intrusive thoughts and images, and engage in various maladaptive coping behaviours, such as seeking excessive medical reassurance, searching for health information online, and checking their body for signs of illness. The DSM-5 proposes two subtypes of IAD: the ‘care-seeking’ subtype for individuals who frequently seek medical care and the ‘care-avoidant’ subtype for individuals who avoid medical care. As IAD is a new disorder, historically, much of the research has focused on samples exhibiting DSM-IV Hypochondriasis (which focused on excessive preoccupation with *having* a serious illness, despite medical reassurance) or excessive and persistent ‘health anxiety’ using dimensional measures (which can encompass current and previous DSM diagnoses and subthreshold symptoms). In this review, we use the term IAD for research on the new DSM-5 diagnosis, and the term health anxiety for the broader literature which informs current understanding of IAD.

## Epidemiology: Prevalence, and Impact

Health anxiety can be conceptualised on a continuum, ranging from mild to severe [2, 3], with IAD at the severe end of this spectrum [1]. The prevalence of health anxiety in the general adult population varies considerably across the literature and is estimated to range between 2.1–13.1% [4, 5]. The prevalence rates of health anxiety are higher in medical settings such as primary care (i.e., general practitioner clinics) and secondary care (i.e., specialised outpatient or inpatient clinics) than in the general community, ranging between 7% and 19.9% [6–8]. Health anxiety has been on the rise over the past three decades in both the community and medical settings [8, 9•]. This increase has been further exacerbated by the COVID-19 pandemic, where health anxiety and fears of COVID-19 were common [10]. If left untreated, health anxiety is chronic, episodic, and can last for years [11, 12]. Unlike depression and anxiety disorders, which tend to show higher rates in females, health anxiety affects men and women equally [13–15]. While the onset of health anxiety typically occurs in early adulthood [1], some research suggests that it may start as early as childhood in some cases [12, 13, 16–18].

Health anxiety causes significant impact and impairment to individuals and society. For example, health anxious individuals report worse self-rated health, more interference with daily activities such as household duties, self-care and mobility, more personal distress, and are at increased risk of early mortality [4, 19–21]. Health anxiety impacts at a societal level; individuals with health anxiety report more absenteeism from work than the general population [22] and higher health care utilisation even compared to individuals with well-defined medical conditions [11]. Although the significant burden of health anxiety on individuals and society is recognised, there are gaps in our understanding of the condition. This review aims to summarise recent literature on health anxiety, and specifically on Illness Anxiety Disorder (where research has studied the new DSM-5 diagnosis), identify research gaps, discuss ongoing debates, and suggest avenues for future research to enhance our understanding of the condition.

## Issues with Diagnosis and Classification

### Diagnostic Criteria

According to the DSM-5, IAD is only diagnosed when a person experiences either no or mild somatic symptoms. If they experience health anxiety with moderate to severe somatic symptoms, they may be diagnosed with Somatic Symptom Disorder (SSD) instead [1]. For an IAD diagnosis, the individual will experience excessive worry about either having or developing a serious medical illness, which persists for six months or more. The individual will be highly vigilant about their personal health status, resulting in various maladaptive coping behaviours (i.e., checking their bodies for signs of illness, excessively searching the internet for health-related information, and making frequent visits to medical settings). Individuals with an SSD diagnosis will experience excessive and persistent thoughts related to the seriousness of their moderate-to severe somatic symptoms, and/or high levels of anxiety about their health or symptoms and dedicate significant time to researching and understanding their symptoms in depth. Their somatic symptoms must also last longer than 6 months but can change across their course. IAD and SSD were put forward to replace DSM-IV Hypochondriasis due to its limited clinical validity, and research suggests that IAD and SSD are more reliable than Hypochondriasis [12]. However, having two separate diagnoses is not clinically useful, as research has found there are few differences in how individuals with health anxiety experience the disorders [12, 23••, 24, 25]. Specifically, the differences that have been reported relate to symptom severity, whereby IAD characterizes individuals with a milder form of health anxiety and SSD represents individuals with a more severe form of health anxiety. Additionally, research has shown that both IAD and SSD respond similarly to psychological treatment [26]. Furthermore, recent research done by our team [25] showed that there were no differences on demographic characteristics, clinical characteristics (i.e., depression and generalized anxiety severity, quality of life related to mental health), the nature and course of health anxiety, or mental health comorbidity, between people who met IAD criteria with no/mild somatic symptoms versus those with moderate-to-severe somatic symptoms. These findings suggest that the distinction in somatic symptom severity between these disorders might be arbitrary [25, 27]. Further research is needed to understand whether restricting IAD to only those with minimal somatic symptoms is clinically useful.

The DSM-5 categorizes IAD into two subtypes: ‘care-seeking’ for individuals that frequently seek medical care, and ‘care-avoidant’ for individuals that frequently avoid medical care. Although researchers have proposed that individuals seek care as a form of reassurance and avoid care out of an overwhelming fear of having a serious disease [28], evidence about these behaviours remains scarce. To date, our team conducted the only study investigating the prevalence of IAD subtypes and found that 25% met criteria for the ‘care-seeking’ subtype, 14% met criteria for the ‘care-avoidant’ subtype, and interestingly, 61% reported fluctuating between seeking and avoiding care. This suggests that there may be a third subtype of IAD which needs further study [12].

### Classification

There has been a long-standing debate about whether health anxiety would be better classified under “Anxiety Disorders” than under its current classification “Somatic Symptom and Related Disorders” due to its shared features and high comorbidity with anxiety disorders, namely Panic Disorder, Obsessive–Compulsive Disorder, and Generalized Anxiety Disorder [29–32]. For example, health anxiety involves both hypervigilance to bodily sensations (a common characteristic in Panic Disorder) and excessive worry (a core feature of Generalized Anxiety Disorder). Research using the DSM-IV Hypochondriasis criteria has shown higher levels of comorbidity with anxiety disorders than comorbid depressive or somatoform disorders [33]. Similarly, substantial comorbidity was found between DSM-5 IAD and other anxiety disorders, particularly Generalized Anxiety Disorder and Panic Disorder [12]. To determine whether IAD should be better classified as an anxiety disorder, future research should investigate whether IAD symptom profiles, course, treatment response and comorbidities more closely align with anxiety disorders compared with other somatic symptom disorders.

## Cognitive and Behavioural Aspects of Health Anxiety

### Theories

Several theories have been proposed to explain the development of health anxiety. The most widely tested theory is based on the cognitive-behavioural model [34] which suggests that individuals with health anxiety tend to misinterpret bodily sensations, often attributing them to serious illness or disease. This catastrophic thinking leads to the adoption of maladaptive safety behaviours, such as seeking excessive medical reassurance, googling of symptoms and, in some cases, avoiding medical settings or individuals who are sick as a way to manage anxiety symptoms. This theoretical model of health anxiety has been subject to empirical investigation, and research broadly supports the components of this model [35].

The other major theory of health anxiety is based on the interpersonal model [36]. This model proposes that anxious and insecure attachments derived from caregivers in early childhood leads to increased maladaptive care-seeking behaviours and reassurance seeking in adulthood [37]. The core tenant of this theory is that insecurely attached individuals seek emotional support via complaints of physical illness and symptoms to reduce the feelings of insecurity. It is important to note that this theory has not been widely tested.

### Cognitive Components

One of the core characteristics of health anxiety is the presence of dysfunctional health beliefs, i.e., the preoccupation with the inaccurate belief that one has, or is in danger of developing, a serious medical illness [38]. These beliefs persist despite appropriate medical evaluation and reassurance [39]. Dysfunctional health-related beliefs comprise of either disease conviction (i.e., the belief one has a feared illness or disease) and/or fear of contracting an illness or disease [40]. Salkovskis and Warwick’s [40] Cognitive-Behavioural Model of health anxiety identifies four types of dysfunctional beliefs central to health anxiety, including the perceived i) likelihood and ii) awfulness of experiencing a health problem, iii) the inability to cope with experiencing a health problem, and (iv) inadequacy of medical resources to treat a health problem [40, 41]. These beliefs are key vulnerability and maintaining factors in health anxiety [42, 43], and are argued to develop in response to aversive learning experiences during childhood or later in life, such as the death or illness of an attachment figure [44].

It is proposed that these beliefs contribute to catastrophic misinterpretations of the significance of health-related stimuli, such as bodily sensations or changes in bodily functions or appearance, leading to intense anxiety [45]. A key contribution to this misinterpretation is an attentional bias towards health-threat related stimuli, which has shown to be strongly associated with health anxiety in a recent systematic review and meta-analysis [46]. Health-anxious individuals have also been shown to experience more frequent and intense illness-related intrusive thoughts [47, 48], and to demonstrate a selective negative interpretation bias of these thoughts or sensations [49].

Further cognitive constructs related to health anxiety include higher levels of anxiety sensitivity, the fear of arousal-related bodily sensations [50, 51], health-related rumination [52], and health-related intolerance of uncertainty which is essentially a reduced capacity to endure the perception of missing important information [53–55].

### Behavioural Components

To manage anxiety and distress triggered by these dysfunctional beliefs and interpretations, health-anxious individuals employ a range of maladaptive coping or safety-seeking behaviours. Each of these behaviours is maladaptive in that although they reduce distress in the short-term, they prevent the disconfirmation of incorrect health-anxious beliefs, thus maintaining the disorder [56]. These typically comprise of reassurance-seeking from external sources (e.g., loved ones or doctors), excessive body checking behaviours (e.g., repeatedly taking blood pressure) and/or avoidance of disease-related cues (e.g., hospitals) [57]. Compulsive and excessive online researching about health worries is a key feature seen in health anxiety, also known as ‘cyberchondria’ [58]. Overutilisation of health services is also considered a core maladaptive coping behaviour in health anxiety [11, 59].

## Risk Factors and Precipitants

Research into precipitants and risk factors for IAD is lacking. Several life experiences have been proposed as precipitants of health anxiety, such as experiencing major life stress, a serious threat to one’s health, witnessing a loved one’s illness, and being exposed to health-threatening information [34, 45]. However, evidence supporting these precipitants is lacking.

No studies have examined risk factors for developing IAD using the new DSM-5 criteria. However, a recent systematic review of studies using dimensional health anxiety measures showed some evidence of a positive association between childhood experiences of illness, intergenerational transmission of health anxiety (including genetic heritability and vicarious observations of parents with health anxiety), childhood traumatic experiences, and the development of health anxiety [60]. However, it is unclear whether these risk factors were specific to health anxiety or general risk factors for internalizing disorders. Of the limited research that has been conducted investigating the intergenerational transmission of health anxiety, a twin study found that 34–37% of health anxiety traits, such as fear of illness and interference with functioning caused by bodily sensations, were related to genetics, whereas the remaining traits were attributed to environmental factors [61].

## Neurobiology

There has been a lack of research examining the neurobiological basis for IAD and health anxiety more broadly. Studies using the emotional stroop task and implicit association test have both showed increased amygdala activation, as well as activity in the rostral anterior cingulate cortex [62] and right posterior parietal cortex and nucleus accumbens [63] in response to body-symptom words between health anxious and healthy controls. In contrast, a recent study found no differences in amygdala activity or any brain activity between patients with health anxiety and healthy controls when exposed to health-related pictures [55]. More research is needed to understand the biological underpinnings of IAD.

## Assessment and Treatment

### Assessment

Dimensional measures of health anxiety are widely used to determine the presence of severe and pathological health anxiety. The three most widely used assessment measures are the Whitley Index [64], Illness Attitude Scale [65], and the Health Anxiety Inventory [66]. All three measures have been psychometrically validated and shown good reliability [66, 67]. A shortened version of the Health Anxiety Inventory [66] and Whitley Index 6 [68] have been developed and widely used in clinical settings due to their practicality and brief nature [69, 70]. However, due to the lack of research in IAD samples, the optimal cut-off scores to detect IAD on these widely used measures are unknown (as studies have validated them against Hypochondriasis criteria).

Diagnostic instruments have also been developed and used to assess IAD. The three most widely used standardized, semi-structured interviews are: The Anxiety and Related Disorder Interview Schedule for DSM-5 – Adult Version [71], the Health Preoccupation Diagnostic Interview [72], and the Structured Clinical Interview for DSM-5 (SCID-5) [73].

### Treatment

The most widely supported evidence based psychological treatment for health anxiety and IAD is Cognitive Behavioural Therapy (CBT). CBT typically consists of strategies including psychoeducation; cognitive restructuring of catastrophic beliefs about health and bodily symptoms; behavioural strategies to reduce hypervigilance to body sensations and compulsive coping behaviours such as body checking, reassurance-seeking, and avoidance; and relapse prevention. Evidence from meta-analyses [74, 75••, 76] shows that CBT is a highly efficacious and cost-effective treatment for health anxiety, with a moderate to large pooled effect size on health anxiety [Hedge’s g = 0.81, 95% CI [0.37, 1.26]; 76] compared to non-CBT controls, with improvements largely maintained over 12–18 months [74]. Evidence also supports the delivery of CBT for health anxiety and IAD online [26, 58, 77], with large effect sizes when delivered in routine care [78, 79]. For example, a non-inferiority trial compared internet-delivered to face-to-face CBT (including health anxious sample with either IAD and SSD) and found no difference in outcome, but with internet-delivered therapy resulting in lower net societal costs [80].

Although less research has been conducted on other psychological therapies for health anxiety, some evidence also supports the use of third-wave therapies for health anxiety such as Mindfulness-Based Cognitive Therapy (MBCT) [81, 82] and Acceptance and Commitment Therapy (ACT) [83, 84], although no studies have tested them in IAD specifically. MBCT treats health anxiety through applying mindfulness techniques to defuse from catastrophic health cognitions, reduce attentional bias towards physiological symptoms, and confront rather than experientially avoid feared body sensations, thus reducing unhelpful avoidance or safety-seeking behaviours [85]. Acceptance and Commitment Therapy (ACT) similarly aims to treat health anxiety by reducing experiential avoidance of distressing illness-related thoughts, feelings, and body sensations, promoting acceptance of these inner states, while clarifying values and encouraging behaviours that contribute to a meaningful life [84]. A recent randomised controlled trial involving ACT delivered online showed large effect sizes in reducing health anxiety at six-month follow up compared to control (d = 0.80, 95% CI 0.38–1.23) [83]. Pharmacotherapy studies are lacking, but two studies into treatments for Hypochondriasis support the use of Fluoxetine in combination with CBT, or alone [86, 87], and another earlier study found Paroxetine yielded similar effectiveness to CBT for hypochondriasis [88]. No pharmacological studies have yet been conducted on the new IAD diagnosis.

## Children and Youth

Cross-sectional studies have shown that children and adolescents can experience severe health anxiety as assessed using dimensional measures [89, 90]. However, research examining the prevalence of health anxiety using diagnostic criteria suggests that very few children meet full diagnostic criteria [18, 91, 92], leading researchers to argue that this could be due to a lack of appropriate descriptions of how severe health anxiety presents in children and youth [93]. No specific CBT treatments have been developed to treat health anxiety or IAD in children or youth [94••] meaning that there are no treatment studies yet conducted in this age group. Further, there have been no studies investigating third wave therapies or pharmacological approaches to treat health anxiety or IAD in young people. More research in children and adolescents is needed, particularly early intervention studies given the chronic and life-long nature of health anxiety when left untreated.

## Conclusions

This review highlights the recent literature on Illness Anxiety Disorder (health anxiety) and identifies current gaps and points for future directions. First, the long-lasting impact of health anxiety on the individual and society is well-documented in the literature. Individuals with health anxiety are debilitated by dysfunctional health beliefs, which, in turn, manifest into maladaptive coping behaviours. There are reliable dimensional measures of health anxiety and diagnostic instruments to assess IAD. CBT is an effective treatment option for this population, in both face-to-face and digital formats. However, further research is needed into third-wave treatments (i.e., MCBT and ACT) and pharmacotherapy in samples with IAD.

Second, there is some evidence of a relationship between illness experiences, intergenerational transmission of health anxiety, and childhood traumatic experiences as risk factors for health anxiety. However, it is unclear whether these factors cause health anxiety.

Third, although the limitations of DSM-IV Hypochondriasis were addressed in the DSM-5 with two new disorders, a debate continues about whether the two disorders should be combined or remain distinct. Early evidence suggests that differences between IAD and SSD are a matter of severity rather than of any distinctive qualitative characteristics, but replication research is needed across varying populations and groups. It is also debated whether IAD should be reclassified into the anxiety disorders category in the next iteration of the DSM, due to its shared characteristics and high comorbidity with other anxiety disorders. Future research should examine the predictive validity of IAD with and without comorbid anxiety in terms of course and treatment response. In addition, there has been little done to investigate the ‘care-seeking’ and ‘care-avoidant’ subtypes of IAD. Future research is necessary to advance knowledge of this condition, particularly if there are differences between these groups in terms of their nature and experience, and response to treatment.

Finally, recent evidence suggests health anxiety may start, at least for some, in childhood and adolescence. However, due to a lack of developmentally appropriate diagnostic criteria for young people, there is a need to investigate how health anxiety presents in children and young people and an urgent need to develop or adapt treatment options that are appropriate for this population.

Overall, the most significant gap in this field is the limited number of studies utilising the current diagnostic criteria for Illness Anxiety Disorder. Addressing this gap is crucial if we are to significantly advance knowledge, improve our understanding of the disorder, intervene early, and develop targeted, effective treatments for the disorder.

## Data Availability

No datasets were generated or analysed during the current study.
